# Association of Subcortical Structural Shapes With Tau, Amyloid, and Cortical Atrophy in Early-Onset and Late-Onset Alzheimer’s Disease

**DOI:** 10.3389/fnagi.2020.563559

**Published:** 2020-10-26

**Authors:** Eun-Chong Lee, Jae Myeong Kang, Seongho Seo, Ha-Eun Seo, Sang-Yoon Lee, Kee Hyung Park, Duk L. Na, Young Noh, Joon-Kyung Seong

**Affiliations:** ^1^School of Biomedical Engineering, Korea University, Seoul, South Korea; ^2^Department of Psychiatry, Gil Medical Center, Gachon University College of Medicine, Incheon, South Korea; ^3^Department of Neuroscience, College of Medicine, Gachon University, Incheon, South Korea; ^4^Neuroscience Research Institute, Gachon University, Incheon, South Korea; ^5^Department of Neurology, Gil Medical Center, Gachon University College of Medicine, Incheon, South Korea; ^6^Department of Neurology, Samsung Medical Center, Sungkyunkwan University School of Medicine, Seoul, South Korea; ^7^Neuroscience Center, Samsung Medical Center, Seoul, South Korea; ^8^Department of Health Science and Technology, GAIHST, Gachon University, Incheon, South Korea; ^9^Department of Artificial Intelligence, Korea University, Seoul, South Korea

**Keywords:** Alzheimer’s disease, subcortical shape analysis, tau, amyloid, cortical thickness, positron emission tomography, magnetic resonance imaging

## Abstract

The objectives of this study were to compare the topographical subcortical shape and to investigate the effects of tau or amyloid burden on atrophic patterns in early onset Alzheimer’s disease (EOAD) and late-onset Alzheimer’s disease (LOAD). One hundred and sixty-one participants (53 EOAD, 44 LOAD, 33 young controls, and 31 older controls) underwent [18F]THK5351 positron emission tomography (PET), [18F]flutemetamol (FLUTE) PET, and 3T MRI scans. We used surface-based analysis to evaluate subcortical structural shape, permutation-based statistics for group comparisons, and Spearman’s correlations to determine associations with THK, FLUTE, cortical thickness, and neuropsychological test results. When compared to their age-matched controls, EOAD patients exhibited shape reduction in the bilateral amygdala, hippocampus, caudate, and putamen, while in LOAD patients, the bilateral amygdala and hippocampus showed decreased shapes. In EOAD, widespread subcortical shrinkage, with less association of the hippocampus, correlated with THK retention and cortical thinning, while in LOAD patients, subcortical structures were limited which had significant correlation with THK or mean cortical thickness. Subcortical structural shape showed less correlation with FLUTE global retention in both EOAD and LOAD. Multiple cognitive domains, except memory function, correlated with the bilateral amygdala, caudate, and putamen in EOAD patients, while more restricted regions in the subcortical structures were correlated with neuropsychological test results in LOAD patients. Subcortical structures were associated with AD hallmarks in EOAD. However, the correlation was limited in LOAD. Moreover, relationship between subcortical structural atrophy and cognitive decline were quite different between EOAD and LOAD. These findings suggest that the effects of Alzheimer’s pathologies on subcortical structural changes in EOAD and LOAD and they may have different courses of pathomechanism.

## Introduction

Alzheimer’s disease (AD) is a neurodegenerative disease associated with cognitive decline. Accumulations of neurofibrillary tangles and beta-amyloid (Aβ) are the two pathologic hallmarks of AD, as well as structural changes in the brain cortices (Jack et al., 2016). Early onset AD (EOAD) is characterized by onset before 65 years of age and accounts for 10–15% of all cases (Panegyres and Chen, 2014; Wattmo and Wallin, 2017). Studies have found that EOAD patients have typical neuropsychological features with more progressive cognitive decline (Seltzer and Sherwin, 1983; Smits et al., 2012) as well as heterogenous neuroimaging findings (Frisoni et al., 2007; Migliaccio et al., 2015).

Compared with LOAD patients, EOAD patients manifest with more widespread tau PET retention and heavier amyloid PET uptake in the brain cortices, and more severe gray matter loss suggesting different etiologies and predisposing factors for EOAD (Frisoni et al., 2007; Choo et al., 2011; Scholl et al., 2017; Wattmo and Wallin, 2017). Like gray matter change, more rapid volumetric declines in several subcortical structures including the caudate, putamen, and thalamus have been highlighted as distinct to LOAD patients (Cho et al., 2013; Pievani et al., 2013). It has also been reported that characteristic symptoms of EOAD such as severe extrapyramidal signs (Chui et al., 1985) and non-memory cognitive dysfunction such as executive function, visuospatial functioning, and attention (Smits et al., 2012) are related to deterioration of the basal ganglia and the thalamus, both of which play major roles in movement symptoms, brain connectivity, and memory function (Hahn et al., 2016; Dipasquale and Cercignani, 2017).

Although there have been previous investigations into the association between tau, Aβ, and atrophy in brain cortices, little is known of the relationship between the well-known AD biomarkers and subcortical structures. Unraveling the effect of tau, Aβ, and atrophy in the subcortical structures may contribute to a better understanding of the pathological mechanisms of AD and the different developmental paths of EOAD and LOAD. We sought to compare topographical changes in the subcortical structures in EOAD and LOAD patients and investigated their association with cortical tau and amyloid global retention, as well as cognitive functions.

## Materials and Methods

### Participants

Ninety-one participants who had been clinically diagnosed with AD dementia (EOAD; *n* = 55, LOAD; *n* = 46) and 66 cognitively normal (CN) participants were prospectively recruited. All participants underwent [^18^F]THK5351 PET scans, [^18^F]flutemetamol (FLUTE) PET scans, and 3.0-Tesla MRI scans at Gachon University Gil Medical Center, from October 2015 to June 2017. Of the 167 participants, 2 patients diagnosed with AD were excluded due to head motion issues during the [^18^F]THK5351 PET scan acquisition. An additional four participants (two AD and two CN) were excluded due to errors with the FreeSurfer software. Thus, data from 161 participants (53 EOAD, 44 LOAD, 33 YC, and 31 OC) were used in the analyses.

All patients with AD dementia met the probable AD criteria as proposed by the National Institute of Neurological and Communicative Disorders and Stroke and the AD and Related Disorders Association (McKhann et al., 1984). Patients with familial AD with autosomal dominant inheritance were excluded. Participants with brain lesions on brain MRI such as intracranial hemorrhage, traumatic brain injury, hydrocephalus, territorial infarction, severe white matter hyperintensity (WMH) or WMH associated with radiation, multiple sclerosis or vasculitis were excluded. Severe WMH was defined as both periventricular WMH as cap or band ≥10 mm and deep WMH ≥25 mm using modified Fazekas visual rating scale (Fazekas et al., 1987; Noh et al., 2014). Secondary causes of dementia were ruled out through serum laboratory tests including for thyroid function, metabolic profile, vitamin B_12_, folate, complete blood counts, and syphilis serology. APOE genotyping was also performed. Clinical interviews with a neurologist or psychiatrist and standardized comprehensive neuropsychological tests were undertaken with all participants.

CN participants were either volunteers from the community or spouses of the patients (age range 44–92, female 45.31%). They had no history of neurologic diseases, psychiatric disorders, abnormalities on neurologic examination, structural lesions on brain MRI such as cerebral infarction, intracranial hemorrhage, traumatic brain injury, hydrocephalus, or severe WMH. Control participants had a clinical dementia rating (CDR) score of 0, and normal cognitive function defined as within 1.5 standard deviations of the age- and education-corrected normative mean on detailed neuropsychological tests. Written informed consent was obtained from each participant, and the Institutional Review Boards of Gachon University Gil Medical Center approved this study.

### Image Data Acquisition and Parcellation

3D T1-MPRAGE (Repetition time: 1,900 ms, echo time: 2.93 ms, flip angle: 8°, pixel bandwidth: 170 Hz/pixel, matrix size: 256 × 208, field of view: 256 mm^2^ × 208 mm^2^, slice thickness: 1.0 mm, voxel size: 1.0 mm^3^ × 1.0 mm^3^ × 1.0 mm^3^, NEX: 1, total acquisition time: 4 min 9 s, GRAPPA acceleration factor: 2 along phase-encoding direction) was acquired with a 3.0T MRI (Verio, Siemens with a Siemens matrix coil). Images were analyzed using FreeSurfer 5.1^1^ to define regions-of-interest (ROIs) in native-space for each participant and to support a correction of gray matter atrophy and white matter spillover in the PET data. In addition, subcortical region parcellation including the left and right of the amygdala, caudate, hippocampus, pallidum, putamen, and thalamus was also conducted using Desikan-Killiany atlas in MNI 152 space. After subcortical segmentation, each subcortical region on the template surface was sampled as a mesh surface with 2,562 vertices and then transformed into its own original surface. This procedure was conducted for every 12 subcortical regions as detailed in a previous study (Chung et al., 2017; Koo et al., 2017).

The pretreatment process for the PET images proceeded according to the process outlined in a previous study (Kang et al., 2017). [^18^F]THK5351 was synthesized and radiolabeled at Gachon University Neuroscience Research Institute. All participants underwent a 20-min emission scan beginning 50 min after 185 MBq of [^18^F]THK5351 was injected intravenously (50–70 min), and a 20-min emission scan 90 min after the intravenous injection of 185 MBq of [^18^F]FLUTE (90–110 min).

### PET Quantification

The quantification process for the [^18^F]THK and [^18^F]FLUTE PET images was conducted using the same methodology outlined in a previous study (Kang et al., 2017). After co-registration of PET images with the corresponding T1 image, region-based partial volume correction was performed. The regional mean values were then calculated and weight-averaged for pre-defined ROIs (Greve et al., 2014, 2016; Kang et al., 2017). Regional standardized uptake value ratios (SUVRs) were calculated using cerebellar gray matter as the reference region for THK images and the pons for FLUTE images (Okamura et al., 2014; Thurfjell et al., 2014; Lockhart et al., 2016). THK5351 global SUVR was calculated based on the AD-related regions including the frontal, parietal, precuneus, occipital, temporal, anterior cingulate, and posterior cingulate, and the cortical composite FLUTE SUVR was calculated based on AD-related regions including the frontal, parietal, lateral temporal, anterior and posterior cingulate cortices (Thurfjell et al., 2014).

### Assessment of Local Shape of Subcortical Structures

To measure the degree of deformation of the subcortical structures, a subcortical mesh was constructed similarly to that described in previous studies (Cho et al., 2011; Chung et al., 2017; Koo et al., 2017). The local shape at every location was then calculated using the surface-based method proposed by Shapira et al. (2008). Weighted sum of the depths at every location was measured in various directions at every location, using the weight as an angle with the normal vector (Chung et al., 2017; Koo et al., 2017). This depth value obtained at 2,562 locations for a single structure reflects local contraction or local shrinkage at that location (i.e., the smaller the value, the more shrinkage caused by atrophy occurred at that position, which expands as the value becomes larger). Therefore, these values were set as the subcortical local shape. This method was undertaken for each of the subcortical regions (amygdala, hippocampus, caudate, pallidum, putamen, and left and right areas of the thalamus).

### Neuropsychological Assessment

All participants underwent the Korean version of the mini-mental state examination (MMSE), CDR, and a detailed neuropsychological function test battery (Kang et al., 2003) including attention, praxis, language, verbal and visual memory, visuospatial function, frontal/executive function, and elements of Gerstmann syndrome. Details of the comprehensive test battery are described in our previous study (Lee et al., 2018) and all scores were calculated as age- and level of education-matched z-scores.

### Statistical Analysis

A pairwise group comparisons comparing EOAD and LOAD, EOAD and YC, LOAD and OC, YC and OC for subcortical structure shape were performed by permutation tests with 5,000 iterations with ANCOVA. For the comparison between EOAD and LOAD, or between YC and OC, the local shape of the subcortical structures were adjusted for sex, years of education, and intracranial volume (ICV). Additionally, for the comparison between EOAD and YC, or between LOAD and OC, the values for the subcortical local shape were adjusted for age, sex, years of education, and ICV. After the permutation test, the Benjamini-Hochberg false discovery rate was used for region-wise multiple comparison (Benjamini and Hochberg, 1995). A *p*-value threshold (0.05) was applied to extract the regions showing significant differences between two groups. If the size of the statistically significant region was smaller than the specific size, it was regarded as noise and removed.

We calculated Spearman’s rank correlation coefficient to identify the relationship between the deformity at every location in the subcortical structures and the three types of neuroimaging findings such as THK5351 SUVR, FLUTE SUVR, and cortical thickness (Han et al., 2013). The vertices with a stronger negative correlation of −0.3 or a stronger positive correlation of 0.3 were then selected. The significance level of each vertex was corrected using the Cluster-Based statistics (Han et al., 2013). Through this process we examined the correlation between THK5351 global SUVR, FLUTE global SUVR, mean cortical thickness and the subcortex structures with age, sex, years of education and ICV as covariates separately within EOAD and LOAD groups. In this process, a *p*-value threshold (0.05) was applied to extract the regions with a significant correlation between the subcortical structure and neuroimaging findings. In cases where the size of the statistically significant region was smaller than the specific size, it was regarded as noise and removed.

To compare the effects of tau, amyloid, and mean cortical thickness on subcortical shape deformity, the relative importance for each factor was calculated using general linear model. For the general linear model, age, sex, education and ICV were used as covariates, and the degree of tau global retention, amyloid global retention, and mean cortical thickness were used as predictors, and local shape was encoded as dependent variable. The r-squared value of the model was calculated from the predictor combinations in all cases in which a specific predictor was included in the general linear model, and the accumulated value was called relative importance and is shown in the Supplementary Figure 1.

Comparisons of the demographic and clinical information were conducted using independent t-test and chi square test followed by Yate’s continuity correction for nominal variables. For volumetric analyses, group comparisons were conducted using independent t-test and analysis of covariance. Correlations between volumes of each subcortical structure and other variables such as THK, FLUTE, cortical thickness, and cognitive function were evaluated using Pearson’s correlation analysis and general linear model. Volume data analysis was conducted with PASW Statistics 23 software (SPSS Inc., Chicago, IL, United States) with significance set at *p* < 0.05 (two-way).

## Results

### Demographics and Clinical Characteristics

Table 1 shows the demographic information and clinical characteristics of the participants. There were no significant differences in MMSE scores or CDR-SOB between EOAD and LOAD patients. Global retention of THK5351 PET and mean cortical thickness were not different between EOAD and LOAD, however, the EOAD group showed significantly higher amyloid global accumulation than the LOAD group (*p* < 0.001).

**TABLE 1:** Demographics and clinical characteristics of the study population.

**Variables**	**EOAD (*n* = 53)**	**YC (*n* = 33)**	**EOAD vs. YC *P* value**	**LOAD (*n* = 44)**	**OC (*n* = 31)**	**LOAD vs. OC *P* value**	**EOAD vs. LOAD *P* value**
Age	60.5 (5.43)	57.54 (7.16)	0.032^a^	77.80 (6.35)	75.32 (5.54)	0.085	< 0.001^a^
Sex (female, *n* [%])	36 (67.9)	13 (39.4)	0.018^a^	33 (75)	16 (51.6)	0.064	0.589
Education (years)	9.42 (3.92)	13.45 (3.52)	< 0.001^a^	7.30 (5.06)	10.68 (5.34)	0.007^a^	0.022^a^
Disease duration (months)	42.83 (20.55)	0.0 (0.0)	< 0.001^a^	52.39 (30.35)	0.0 (0.0)	< 0.001^a^	0.171
MMSE	16.08 (7.14)	28.76 (1.15)	< 0.001^a^	17.56 (7.04)	27.13 (2.38)	< 0.001^a^	>0.999
CDR-SOB	5.62 (3.90)	0.00 (0.00)	< 0.001^a^	5.11 (3.20)	0.00 (0.00)	< 0.001^a^	0.066
APOE genotype (positive, *n* [%])	25 (47.2)	9 (27.3)	0.066	23 (52.3)	7 (22.6)	0.010^a^	0.617
Mean CTh	2.29 (0.16)	2.49 (0.08)	< 0.001^a^	2.34 (0.13)	2.44 (0.10)	< 0.001^a^	0.060
THK global retention	2.38 (0.51)	1.46 (0.20)	< 0.001^a^	2.23 (0.39)	1.77 (0.24)	< 0.001^a^	0.110
FLUTE global retention	1.07 (0.18)	0.39 (0.06)	< 0.001^a^	0.89 (0.21)	0.43 (0.15)	< 0.001^a^	< 0.001^a^

*Abbreviations: EOAD, early-onset Alzheimer’s disease; LOAD, late-onset Alzheimer’s disease; YC, young control; OC, old control; MMSE, mini-mental state examination; CDR-SOB, clinical dementia rating-sum of boxes; APOE, apolipoprotein ε4 allele; CTh, cortical thickness; SUVR, standard uptake value ratio. Data is presented as mean (standard deviation) for continuous variables and number (%) for nominal variables. MMSE and CDR-SOB were compared between AD and control after adjusting for age and years of education and compared between EOAD and LOAD after adjusting for years of education. *P*-values refer to two-sample *t*-test for continuous variables and Chi square test followed by Yate’s continuity correction for nominal variables. ^*a*^*P* < 0.05.*

### Comparison of Subcortical Structures

**FIGURE 1:**
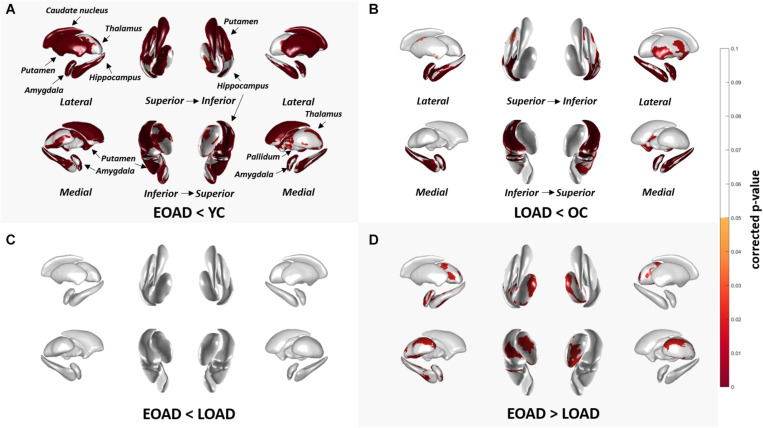
Group comparison of shape of subcortical structures. **(A,B)** Group-wise comparison of the subcortical local shape adjusted with age, sex, education, ICV; **(C,D)** sex, education, ICV was adjusted. Corrected *p*-values after FDR multiple correction for each 12 structures (left and right amygdala, hippocampus, caudate, pallidum, putamen, and thalamus) were mapped. Abbreviations: EOAD, early-onset Alzheimer’s disease; LOAD, late-onset Alzheimer’s disease; YC, young control; OC, old control.

Figure 1 shows the results of group comparisons of the subcortical structural shapes. The EOAD group showed widespread subcortical shape difference in the bilateral amygdala, caudate, hippocampus, putamen, thalamus and the right pallidum relative to YC. The LOAD group showed significant regional shape difference in the bilateral amygdala, hippocampus, and putamen compared to OC. Comparisons of the subcortical structures between the EOAD and LOAD groups showed shrinkage in the bilateral thalamus, left amygdala and left hippocampus in LOAD group.

Volume analysis of the subcortical structures between the groups are presented in Supplementary Table 1. EOAD patients showed reduced volume in the bilateral amygdala, hippocampus, caudate, putamen, and the left thalamus compared to YC. LOAD patients showed reduced volumes in the bilateral amygdala and hippocampus compared to OC.

### Correlation Between THK5351, FLUTE, Cortical Thickness, and Subcortical Structures

**FIGURE 2:**
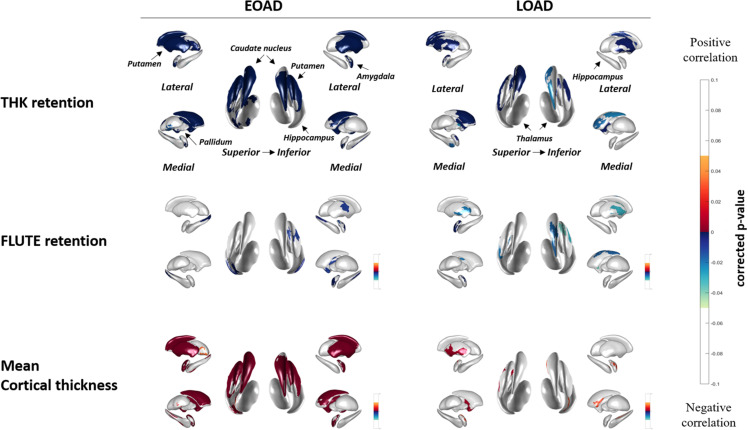
Spearman correlation between subcortical local shape and THK5351 retention, FLUTE global retention, and mean cortical thickness in EOAD and LOAD, adjusting with age, sex, years of education, and intracranial volume (ICV) in each EOAD and LOAD group. The mapped *p*-values are multiple corrected using cluster-based statistics. Abbreviations: EOAD, early onset Alzheimer’s disease; LOAD, late-onset Alzheimer’s disease.

Figure 2 shows the correlation between THK global retention, FLUTE global retention, mean cortical thickness, and subcortical shape in the EOAD and LOAD groups. In EOAD patients, as THK global retention increased, subcortical structure showed contraction significantly in the bilateral amygdala, caudate, putamen, hippocampus, left pallidum and the left thalamus. In LOAD patients, as THK global retention increased, subcortical shape decreased in the bilateral caudate, putamen, left pallidum and the left amygdala and hippocampus.

As FLUTE global retention increased, subcortical shape deformity significantly increased in the bilateral hippocampus and the right amygdala and left putamen in EOAD. LOAD patients showed significant subcortical shape deformity in the bilateral putamen, the left amygdala and hippocampus, and the right caudate as FLUTE increased. There was no subcortical structure with positive correlation with THK or FLUTE global retention in EOAD or LOAD patients. Mean cortical thickness was positively associated with subcortical structure shape in the bilateral amygdala, caudate, putamen, and the left hippocampus in EOAD.

Supplementary Figure 1 shows association between THK, FLUTE global retention, cortical thickness and subcortical structures, which was analyzed using the general linear model. In EOAD group, similar to the results of our main correlation analyses, tau and mean cortical thickness were found to have great importance in the bilateral caudate and putamen, whereas amyloid showed little importance. On the other hand, in the LOAD patient group, the importance was higher in correlation with tau in both caudate. And mean cortical thickness was found to have importance than other variables in predicting the local shape in the left putamen.

Supplementary Figure 2 shows the voxel-wise analyses results to evaluate the association between subcortical structural volume and THK or FLUTE global retention. In EOAD, decreased volumes of the amygdala, caudate, putamen had association with THK retention in the nearly whole association cortices. Hippocampal volume was associated with THK retention in the bilateral medial temporal region and the right precuneus and parietal regions in EOAD. The hippocampus was the only region with significant correlation with FLUTE global retention in EOAD. In LOAD patients, no subcortical structural volume showed significant correlation with THK or FLUTE global retention in voxel-wise analyses.

Volumetric correlation between THK global retention, FLUTE global retention, mean cortical thickness, and subcortical structures are presented in Supplementary Table 2. In EOAD patients, THK, FLUTE, and cortical thickness correlated with volumes of the subcortical structures except for pallidum. In LOAD patients, no correlation was found between THK, FLUTE, and mean cortical thickness and subcortical volume.

### Correlation Between Cognitive Function and Subcortical Structures

The neuropsychological test results are presented in Table 2.

**TABLE 2:** Neuropsychological test results.

	**EOAD (*n* = 53)**	**YC (*n* = 33)**	**EOAD vs. YC *P* value**	**LOAD (*n* = 44)**	**OC (*n* = 31)**	**LOAD vs. OC *P* value**	**EOAD vs. LOAD *P*-value**
**Attention**							
Digit Span Forward	−0.631.50	0.730.93	< 0.001^a^	0.091.07	0.761.03	0.010^a^	0.020^a^
Digit Span Backward	−1.571.52	0.141.28	< 0.001^a^	−0.561.28	0.111.14	0.024^a^	0.001^a^
**Language and related function**							
K-BNT	−2.112.61	0.051.00	< 0.001^a^	−1.901.59	0.030.91	< 0.001^a^	0.774
**Visuospatial function**							
RCFT copy	−5.565.82	0.580.57	< 0.001^a^	−0.511.86	0.340.74	0.010^a^	< 0.001^a^
**Memory**							
SVLT, immediate recall	−2.281.29	−0.131.02	< 0.001^a^	−1.550.97	−0.050.84	< 0.001^a^	0.004^a^
SVLT, delayed recall	−2.560.83	−0.130.95	< 0.001^a^	−1.960.59	0.100.97	< 0.001^a^	< 0.001^a^
SVLT, recognition	−2.761.51	−0.121.50	< 0.001^a^	−1.501.31	0.000.83	< 0.001^a^	< 0.001^a^
RCFT, immediate recall	−1.940.74	0.750.92	< 0.001^a^	−1.200.76	0.411.14	< 0.001^a^	< 0.001^a^
RCFT, delayed recall	−2.220.84	0.701.01	< 0.001^a^	−1.460.77	0.461.03	< 0.001^a^	< 0.001^a^
RCFT, recognition	−2.241.52	0.221.04	< 0.001^a^	−1.731.62	−0.210.98	< 0.001^a^	0.194
**Frontal executive function**							
COWAT, animal	−2.141.01	−0.221.07	< 0.001^a^	−1.720.94	−0.240.92	< 0.001^a^	0.039^a,b^
COWAT, supermarket	−1.810.96	0.050.94	< 0.001^a^	−1.240.96	0.020.98	< 0.001^a^	0.005^a^
COWAT, phonemic total	−1.311.36	0.261.04	< 0.001^a^	−0.870.96	0.131.00	< 0.001^a^	0.092
Stroop test, color reading	−2.391.30	0.130.75	< 0.001^a^	−0.971.05	0.111.15	< 0.001^a^	< 0.001^a^
TMT-A	−7.7711.75	0.590.77	< 0.001^a^	−1.903.81	−0.171.93	0.018^a^	0.001^a^
TMT-B	−8.366.19	0.011.00	< 0.001^a^	−2.922.32	−0.091.23	< 0.001^a^	< 0.001^a^

*All data are z-scores derived on age- and education-adjusted norms and presented as mean ± standard deviation. Comparisons between EOAD and LOAD were adjusted for clinical dementia rating-sum of boxes. ^*a*^*P* < 0.05; ^*b*^insignificant after region-wise correction for multiple comparisons. Abbreviations: EOAD, early onset Alzheimer’s disease; YC, young control; LOAD, late-onset Alzheimer’s disease; OC, old control; K-BNT, Korean version of the Boston naming test; RCFT, Rey-Osterrieth complex figure test; SVLT, Seoul verbal learning test; COWAT, controlled oral word association test; TMT-A/B, trail making test type A/B.*

Patients with EOAD had significantly worse performance in digit span forward and backward, Rey-Osterrieth complex figure test (RCFT) copy memory test other than RCFT recognition, controlled oral word association test supermarket, Stroop test color reading, trail making test type A and B compared to LOAD patients.

**FIGURE 3:**
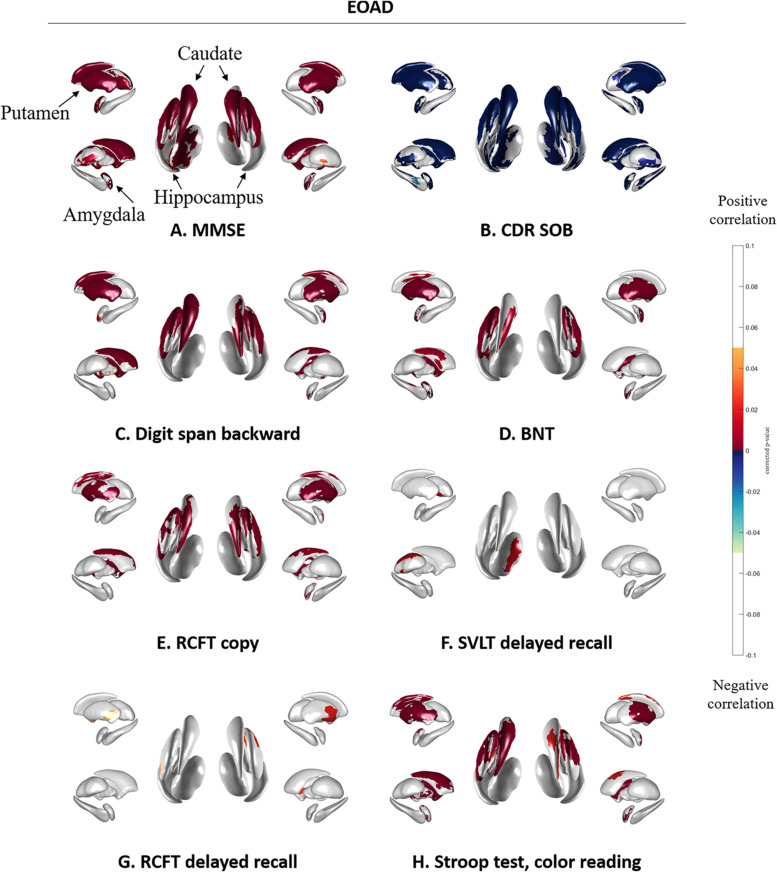
Spearman correlation between neuropsychological test results and subcortical local shape in EOAD group adjusted with intracranial volume. **(A)** MMSE, **(B)** CDR-SOB, **(C)** digit span backward score in attention, **(D)** BNT score in language, **(E)** RCFT copy score in visuospatial, **(F)** SVLT delayed recall score in memory, **(G)** RCFT delayed recall score in memory, and **(H)** color reading test score in frontal/executive function. The mapped *p*-values are multiple corrected using cluster-based statistics. Abbreviations: EOAD, early onset Alzheimer’s disease; MMSE, mini-mental state examination; CDR-SOB, clinical dementia rating-sum of boxes.

Figure 3 shows the association between subcortical shapes and neuropsychological test scores in the EOAD patients. MMSE scores were correlated with the bilateral amygdala, caudate, putamen, and thalamus and the left hippocampus (Figure 3A). CDR-SOB was correlated with the bilateral amygdala, hippocampus, caudate, putamen and thalamus (Figure 3B). Digit span backward was correlated with the bilateral amygdala, caudate, and putamen and the left pallidum (Figure 3C). BNT was correlated with the bilateral amygdala and putamen and the left caudate and hippocampus (Figure 3D). RCFT copy test was correlated with the bilateral caudate and putamen and right amygdala (Figure 3E). SVLT delayed recall showed correlation with the left thalamus (Figure 3F) and RCFT delayed recall showed association with the bilateral putamen (Figure 3G). Executive function measured by Stroop test color reading was correlated with the bilateral amygdala, caudate, and putamen and the left pallidum (Figure 3H).

**FIGURE 4:**
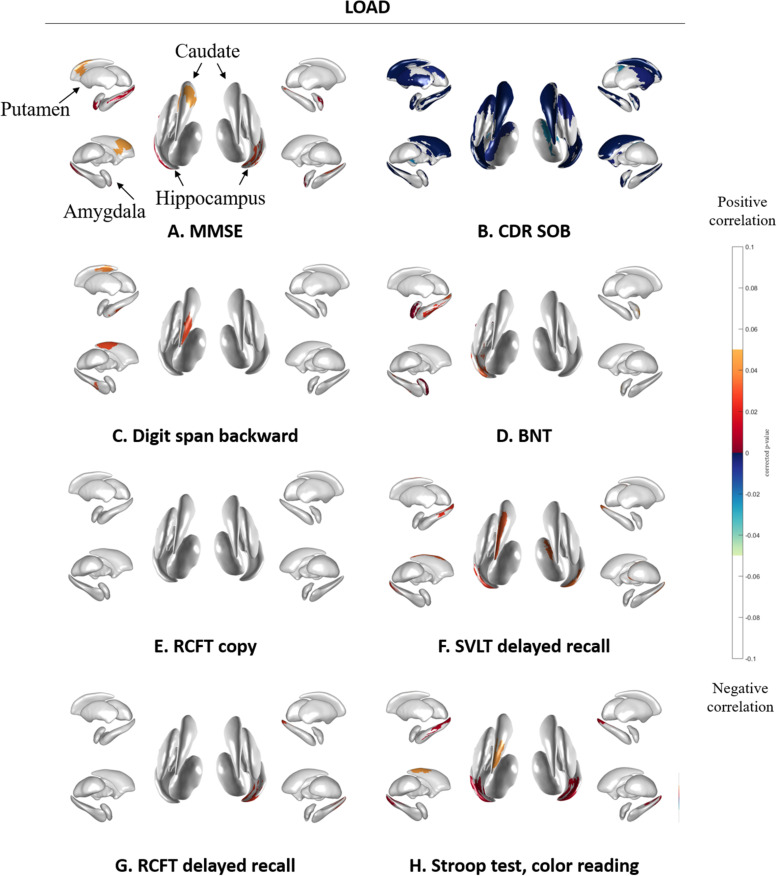
Spearman correlation between neuropsychological test results and subcortical local shape in LOAD group adjusted with intracranial volume. **(A)** MMSE, **(B)** CDR-SOB, **(C)** digit span backward score in attention, **(D)** BNT score in language, **(E)** RCFT copy score in visuospatial, **(F)** SVLT delayed recall score in memory, **(G)** RCFT delayed recall score in memory, and **(H)** color reading test score in frontal/executive function. The mapped *p*-values are multiple corrected using cluster-based statistics. Abbreviations: LOAD, late onset Alzheimer’s disease; MMSE, mini-mental state examination; CDR-SOB, clinical dementia rating-sum of boxes.

Figure 4 shows the association between subcortical shape and neuropsychological test scores in LOAD patients. MMSE scores were correlated with the bilateral amygdala, hippocampus, and putamen and the left caudate (Figure 4A) and CDR-SOB was correlated with the bilateral amygdala, caudate, hippocampus, putamen, thalamus, and the left pallidum (Figure 4B). Digit span backward was correlated with the left caudate and hippocampus (Figure 4C). BNT was correlated with the left amygdala and hippocampus (Figure 4D). RCFT copy showed no association with subcortical structural shape (Figure 4E). SVLT delayed recall was correlated with the bilateral hippocampus and the left caudate, and the right thalamus (Figure 4F), and RCFT delayed recall was correlated with the right hippocampus (Figure 4G). Stroop test color reading was correlated with the bilateral hippocampus and the left caudate (Figure 4H). Results of the correlation analysis between volumes of the subcortical structures and the detailed neuropsychological test results were presented in Supplementary Tables 3 and 4.

## Discussion

In this study, we evaluated the degree of subcortical structural shape deformity in EOAD and LOAD patients, in terms of association with tau and amyloid global retention, cortical thickness, and neuropsychological test results. We observed greater subcortical shape deformity in EOAD and more limited deformation especially in the amygdala and hippocampus in LOAD patients, compared to their age-matched controls. The associations between subcortical structural shape and THK global retention, mean cortical thickness, or cognitive functions were also more prominent in EOAD compared to LOAD patients.

Subcortical shape differed according to the age of onset. Compared to each age-matched control group, EOAD patients exhibited shape deformation in nearly all the subcortical structures, including the bilateral amygdala, hippocampus, caudate, putamen, and thalamus. Meanwhile, LOAD patients manifested decreased shapes in the more restricted region such as the amygdala, hippocampus, and putamen. We also found that the amygdala, hippocampus and thalamus were the major regions that differed in the direct group comparison with more shrinkage in LOAD patients than in EOAD patients. This observation is consistent with previous studies that have shown greater hippocampal atrophy in LOAD compared to EOAD (Frisoni et al., 2007). It was also previously found that EOAD patients mapped with subcortical deformity in wider regions including the amygdala, hippocampus, and putamen compared to LOAD patients with subcortical deformity mapped in the amygdala and hippocampus only, which was also reflected in our findings (Cho et al., 2013). It has been previously reported that EOAD patients show more rapid declines in volume in the bilateral caudate, putamen, and thalamus compared to LOAD (Cho et al., 2013). In another study, researchers found putamen (dorsal striatum) atrophy in EOAD and atrophy in the nucleus accumbens (the ventral striatum) in LOAD patients (Pievani et al., 2013). The limbic area including the hippocampus and amygdala is well known to be affected in the initial stages and the basal ganglia and thalamus are affected in the later stages of LOAD (Braak and Braak, 1991). Our observations may be indicative of a dorsal atrophic pattern of the subcortical structures in EOAD and predominant hippocampal volume loss in LOAD (Pievani et al., 2013).

In terms of tau, amyloid, and cortical thickness, broad regions of the subcortical shape deformation in the caudate and putamen were associated with THK global retention and mean cortical thickness in EOAD patients. There was association with relatively low effect sizes between subcortical shape and FLUTE global retention in both groups. These results were expected because the mean cortical thickness and tau retention reportedly proceeds in a relatively parallel manner (Whitwell et al., 2008, 2018) compared to amyloid retention (Thal et al., 2002). A/T/N classification systems for AD biomarkers note a specific temporal ordering where Aβ (A) precedes tau (T) and neurodegeneration (N), which correlate with clinical symptoms (Jack et al., 2016). Neurofibrillary tangle retention is also known to accumulate to a higher degree in most of the cortical regions sparing the primary sensorimotor cortices in EOAD and in the confined regions including the medial temporal and lateral parietal regions in LOAD (Scholl et al., 2017) compared to age-matched controls. Our results appear to support these past findings and provide evidence that the dorsal subcortical structures such as the caudate and putamen are affected more in EOAD compared to LOAD and it is associated with tau global retention and cortical thinning more significantly than amyloid global accumulation. It was interesting result that amyloid global retentions were correlated with hippocampal shapes in EOAD. APOE e4 genotype might be associated with this correlation. Further study would be needed to investigate this issue.

Subcortical structures correlated with the neuropsychological test results, with greater association found more consistently in EOAD than LOAD patients. Specifically, attention, language, visuospatial function, frontal/executive function, and global cognition was correlated with subcortical shapes in most regions of the caudate and putamen. This corresponds the known function of the caudate and putamen (Koziol and Budding, 2009). The striatum, including the caudate nucleus and putamen, receives inputs from most of the major cortical regions including motor, sensory, perceptual and association, frontal executive, and affective and motivational regions (Koziol and Budding, 2009). The head of the caudate nucleus is known to receive inputs from the dorsolateral prefrontal cortex, while the tail of the caudate receives projections from the parietal cortex, and the putamen receives inputs from the motor cortex (Koziol and Budding, 2009). Regarding the circuitry of the striatum, our results indicate that the subcortical structures may associate with cortical region-specific cognitive impairment in EOAD patients. Contrary to EOAD patients, LOAD patients have shown more limited associations: language impairment was associated with shapes in the left amygdala and hippocampus, verbal memory impairment was associated with shapes in the bilateral hippocampus and the left caudate, and visual memory impairment was associated with shapes in the right hippocampus with lower levels of significance. A previous study found that EOAD patients showed more rapid cognitive decline as determined by the MMSE and frontal and total scores in the comprehensive neuropsychological test battery when compared to LOAD patients (Cho et al., 2013). Memory impairment is known to be a predominant phenotype of LOAD and non-memory impairment, including visuospatial dysfunction and apraxia, is predominant in EOAD (Koedam et al., 2010). The present findings are generally in accordance with these previous reports (Koedam et al., 2010; Cho et al., 2013) showing region-specific associations between subcortical structures and neuropsychological test results in the EOAD and LOAD groups.

We note several limitations to this study. The participants were recruited from a single memory clinic in a tertiary hospital, which may limit generalizability of the results. The lack of information on genetic mutations is also limiting because cognitive function and imaging biomarkers can vary according to genetic differences, especially for comparisons between EOAD and LOAD patients. Although we excluded participants with familial AD with autosomal dominant inheritance, the possibility which participants with genetic mutation could not excluded completely as we did not perform PSEN 1, APP or PSEN2 gene tests to all participants. Another major limitation is the known off-target binding of the tracer used in this study. [18F]THK5351, a first generation tau tracer, is known to bind to monoamine oxidase-B (Ng et al., 2017), and thus can be reactive to both neurofibrillary tangles and reactive astrocytes (Harada et al., 2018). Due to its limited utility as a sole biomarker for tau retention, our results should thus be interpreted with some degree of caution. However, we believe our study remains the first to evaluate subcortical structural shapes in EOAD and LOAD patients, using the three major imaging biomarkers tau PET, amyloid PET, and MRI which can provide valuable evidence of *in vivo* findings in AD.

We observed pattern differences in subcortical structural shape between EOAD and LOAD. Widespread subcortical atrophy was associated with tau and cortical thinning in most of the cognitive domains, except for memory, in EOAD. In contrast, smaller subcortical regions were associated only with THK retention and restricted cognitive impairment including memory in LOAD. These results may be indicative of a different underlying pathomechanism between EOAD and LOAD. In addition, the imaging results are in close agreement with the known progression of the A/T/N *in vivo* biomarkers and differences in cognitive function in both groups. The employment of all available imaging biomarkers and age of onset in AD studies may be helpful in more clearly elucidating the association between changes in subcortical regions and other AD biomarkers.

## Data Availability Statement

The data set generated and/or analyzed using the current study is available from the corresponding author, YN on reasonable request.

## Ethics Statement

The studies involving human participants were reviewed and approved by Institutional Review Boards of Gachon University Gil Medical Center. The patients/participants provided their written informed consent to participate in this study.

## Author Contributions

YN and J-KS conceptualized and designed the study. E-CL and JMK drafted the manuscript. JMK, H-ES, S-YL, KHP, DLN, and YN acquired the data. E-CL, JMK, and SS analyzed the data. J-KS took part in methodology. YN was in charge of funding acquisition and resources. YN and J-KS revised the manuscript for intellectual content. All authors reviewed and approved for publication.

## Conflict of Interest

The authors declare that the research was conducted in the absence of any commercial or financial relationships that could be construed as a potential conflict of interest.

## Footnotes

1 www.surfer.nmr.mgh.harvard.edu
