# Supplementary material for: Association of Subcortical Structural Shapes With Tau, Amyloid, and Cortical Atrophy in Early-Onset and Late-Onset Alzheimer’s Disease
Source: Front Aging Neurosci. 2020 Oct 26;12:563559. doi: 10.3389/fnagi.2020.563559 (PMC7650820; doi:10.3389/fnagi.2020.563559)
Supplement: Supplementary file 1 [file Data_Sheet_1.PDF]

Table S1. Volumes of the subcortical structures

	EOAD (n=53)	YC (n=33)	EOAD vs. YC P value	LOAD (n=44)	OC (n=31)	LOAD vs. OC P value	EOAD vs. LOAD P value
Amygdala L	1263 ± 251	1801 ± 224	<0.001 [a]	1125 ± 248	1589 ± 234	<0.001 [a]	0.001 [a]
Amygdala R	1268 ± 222	1833 ± 177	<0.001 [a]	1207 ± 251	1653 ± 206	<0.001 [a]	0.064
Hippocampus L	3065 ± 469	4366 ± 314	<0.001 [a]	2820 ± 444	3719 ± 389	<0.001 [a]	0.001 [a]
Hippocampus R	3149 ± 551	4471 ± 328	<0.001 [a]	2903 ± 521	3867 ± 324	<0.001 [a]	0.007 [a]
Caudate L	3013 ± 416	3493 ± 419	<0.001 [a]	3003 ± 423	3115 ± 345	0.229	0.754
Caudate R	3119 ± 419	3579 ± 370	<0.001 [a]	3226 ± 382	3287 ± 399	0.503	0.251
Pallidum L	1686 ± 203	1792 ± 217	0.024 [a]	1606 ± 224	1685 ± 201	0.121	0.045 [a, b]
Pallidum R	1479 ± 187	1554 ± 172	0.065	1426 ± 185	1424 ± 160	0.967	0.137
Putamen L	4692 ± 730	5631 ± 558	<0.001 [a]	4417 ± 698	5026 ± 707	<0.001 [a]	0.020 [a, b]
Putamen R	4420 ± 611	5171 ± 571	<0.001 [a]	4334 ± 555	4824 ± 655	0.001 [a]	0.238
Thalamus L	5966 ± 674	6496 ± 501	0.001 [a]	5649 ± 637	5835 ± 547	>0.191	0.011 [a]
Thalamus R	6206 ± 775	6608 ± 635	0.014 [a]	5770 ± 632	6016 ± 473	0.071	0.002 [a]

Abbreviation: EOAD, early-onset Alzheimer's disease; YC, young control; LOAD, late-onset Alzheimer's disease; OC, old control; L, left; R, right

Volumes are expressed in mm^3. Data are presented as mean ± standard deviation.

[a] $P < 0.05$; [b] insignificant after region-wise correction for multiple comparisons

Table S2. Correlation between THK global retention, FLUTE cortical retention, mean cortical thickness, and subcortical volume loss

	Amygdala L	Amygdala R	Hippocampus L	Hippocampus R	Caudate L	Caudate R	Pallidum L	Pallidum R	Putamen L	Putamen R	Thalamus L	Thalamus R
In EOAD												
THK global SUVR	-.625	-.605	-.644	-.593	-.396	-.345	-.163	-.050	-.538	-.493	-.294	-.192
	<.001[a]	<.001[a]	<.001[a]	<.001[a]	<.001[a]	<.001[a]	.044[a, b]	.537	<.001[a]	<.001[a]	<.001[a]	.017[a]
FLUTE cortical SUVR	-.660	-.696	-.728	-.702	-.311	-.294	-.150	-.015	-.472	-.471	-.277	-.207
	<.001[a]	<.001[a]	<.001[a]	<.001[a]	<.001[a]	<.001[a]	.063	.850	<.001[a]	<.001[a]	.001[a]	.010[a]
Mean cortical thickness	.647	.655	.587	.551	.374	.343	.122	.022	.580	.577	.202	.105
	<.001[a]	<.001[a]	<.001[a]	<.001[a]	<.001[a]	<.001[a]	.132	.788	<.001[a]	<.001[a]	.012[a]	.196
In LOAD												
THK global SUVR	-.253	-.023	-.249	-.051	-.196	-.129	-.177	-.071	-.306	-.283	.079	.045
	.115	.886	.122	.754	.226	.429	.274	.661	.055	.077	.626	.784
FLUTE cortical SUVR	-.322	-.112	-.289	-.196	-.170	-.166	-.090	.171	-.416	-.447	-.042	-.225
	.042[a, b]	.493	.071	.225	.294	.305	.583	.291	.008[a, b]	.004[a, b]	.797	.163
Mean cortical thickness	.355	.232	.181	.167	.053	.161	-.140	-.220	.300	.148	-.146	-.079
	.024[a, b]	.150	.263	.303	.746	.321	.390	.173	.060	.362	.370	.627

Abbreviation: EOAD, early-onset Alzheimer's disease; LOAD, late-onset Alzheimer's disease; L, left; R, right; SUVR, standardized uptake value ratio
Pearson's correlation analysis after adjusting age, sex, years of education, and intracranial volume was used and data are coefficient *r* and p value
[a] $P < 0.05$; [b] insignificant after region-wise correction for multiple comparisons

Table S3. Correlation between the neuropsychological test results and subcortical volume loss in patients with EOAD

	Amygdala L	Amygdala R	Hippocampus L	Hippocampus R	Caudate L	Caudate R	Pallidum L	Pallidum R	Putamen L	Putamen R	Thalamus L	Thalamus R
Attention												
Digit Span Forward	.233	.257	.047	.120	.329	.211	.390	.327	.403	.459	.019	-.044
	.159	.120	.781	.473	.044[a,b]	.205	.016[a]	.045[a,b]	.012[a]	.004[a]	.911	.791
Digit Span Backward	.240	.391	.123	.166	.283	.128	.173	.132	.415	.429	.153	.002
	.146	.015[a]	.461	.319	.085	.444	.300	.428	.010[a]	.007[a]	.358	.989
Language and related function												
K-BNT	.314	.280	.223	.251	.395	.314	.148	.215	.461	.507	.076	-.057
	.055	.089	.178	.128	.014[a,b]	.055	.376	.194	.004[a]	.001[a]	.649	.734
Visuospatial function												
RCFT copy	.094	.306	-.119	-.040	.172	.064	.016	.142	.259	.324	-.237	-.363
	.574	.062	.478	.810	.302	.703	.926	.396	.116	.047[a,b]	.151	.025
Memory												
SVLT, immediate recall	.404	.295	.389	.303	.437	.201	.227	.139	.476	.422	.285	.106
	.012[a,b]	.073	.016[a,b]	.065	.006[a]	.225	.170	.405	.003[a]	.008[a]	.083	.527
SVLT, delayed recall	.163	.017	.387	.194	.124	-.027	-.078	.054	.117	.017	.229	-.011
	.327	.918	.016	.243	.460	.873	.641	.748	.483	.918	.166	.950
SVLT, recognition	.351	.220	.447	.265	.197	.045	-.022	-.044	.290	.197	.326	.182
	.031[a,b]	.185	.005[a,b]	.108	.235	.788	.894	.792	.077	.235	.046[a,b]	.274
RCFT, immediate recall	.333	.216	.218	.252	.255	.122	-.030	-.003	.235	.201	.109	-.108
	.041[a,b]	.192	.188	.126	.122	.465	.857	.986	.155	.227	.516	.517
RCFT, delayed recall	.335	.228	.280	.374	.189	.110	.094	.066	.276	.251	.207	.049
	.040[a,b]	.169	.089	.021[a,b]	.255	.511	.574	.694	.093	.129	.213	.772
RCFT, recognition	.200	.051	.297	.234	-.014	-.102	.041	.047	.096	<.001	.141	.085
	.228	.762	.070	.157	.932	.541	.809	.781	.565	>.999	.399	.612
Frontal executive function												
COWAT, animal	.446	.419	.409	.306	.378	.249	.034	-.051	.556	.489	.191	.069
	.005[a]	.009[a]	.011[a]	.062	.019[a,b]	.132	.840	.762	<.001[a]	.002[a]	.250	.680
COWAT, supermarket	.121	.221	.021	-.030	.162	.015	-.212	-.289	.123	.064	.089	.011
	.469	.182	.902	.857	.332	.930	.201	.079	.464	.705	.595	.947
COWAT, phonemic total	.147	.313	-.077	.019	.174	.132	-.077	-.127	.242	.285	-.035	-.115
	.377	.056	.645	.910	.297	.430	.645	.449	.143	.083	.834	.491
Stroop test, color reading	.298	.355	.155	.152	.308	.093	.280	.210	.459	.392	.222	.166
	.070	.029[a,b]	.352	.363	.060	.579	.089	.207	.004[a,b]	.015[a,b]	.181	.320
TMT-A	.255	.318	.122	.095	.402	.272	.198	.269	.424	.416	.141	.009
	.122	.052	.465	.572	.012[a,b]	.098	.234	.103	.008[a,b]	.009[a,b]	.397	.957
TMT-B	.174	.173	-.153	-.097	.296	.171	-.033	.115	.307	.302	-.118	-.278
	.295	.300	.359	.564	.071	.304	.845	.492	.061	.066	.479	.091
Global cognition												
MMSE	.487	.524	.386	.388	.469	.242	.329	.179	.595	.555	.308	.197
	.002[a]	.001[a]	.017[a]	.016[a]	.003[a]	.144	.044[a,b]	.281	<.001[a]	<.001[a]	.060	.237
CDR-SOB	-.465	-.550	-.296	-.303	-.286	-.302	-.196	-.043	-.442	-.509	-.218	-.223
	.003[a]	<.001[a]	.071	.064	.081	.065	.239	.797	.005[a]	.001[a]	.189	.178

Abbreviation: EOAD, early-onset Alzheimer's disease; L, left; R, right; K-BNT, Korean version of the Boston naming test; RCFT, Rey-Osterrieth complex figure test; SVLT, Seoul verbal learning test; COWAT, controlled oral word association test; TMT-A/B, trail making test type A/B; MMSE, mini-mental status examination; CDR-SOB, clinical dementia rating-sum of boxes

Pearson's correlation analysis after adjusting intracranial volume was used and data are coefficient *r* and p value

[a] $P < 0.05$; [b] insignificant after region-wise correction for multiple comparisons

Table S4. Correlation between the neuropsychological test results and subcortical volume loss in patients with LOAD

	Amygdala L	Amygdala R	Hippocampus L	Hippocampus R	Caudate L	Caudate R	Pallidum L	Pallidum R	Putamen L	Putamen R	Thalamus L	Thalamus R
Attention												
Digit Span Forward	.312	.035	.206	-.191	.237	.188	.099	-.088	.272	.190	-.288	-.401
	.082	.849	.257	.295	.192	.303	.590	.634	.132	.299	.110	.023[a,b]
Digit Span Backward	.456	.168	.488	.222	.357	.270	.195	-.016	.435	.274	-.023	-.036
	.009 [a,b]	.359	.005 [a,b]	.222	.045 [a,b]	.135	.286	.929	.013[a,b]	.129	.900	.844
Language and related function												
K-BNT	.415	.237	.070	.093	-.130	-.249	-.310	-.285	-.037	-.104	-.337	-.424
	.018 [a,b]	.192	.703	.614	.480	.169	.084	.113	.840	.569	.060	.015 [a,b]
Visuospatial function												
RCFT copy	.054	-.191	.096	.001	.212	.003	.176	-.001	.312	.077	-.220	-.132
	.771	.296	.602	.997	.244	.986	.334	.997	.082	.676	.226	.471
Memory												
SVLT, immediate recall	.158	.027	.229	.057	.121	-.018	.003	-.063	.056	-.058	-.228	-.166
	.386	.885	.207	.756	.510	.921	.989	.733	.762	.751	.210	.364
SVLT, delayed recall	.045	-.097	.321	-.008	.203	.067	-.228	-.065	-.105	-.185	-.055	.001
	.808	.598	.073	.966	.265	.716	.210	.725	.566	.310	.764	.996
SVLT, recognition	.361	.254	.456	.229	.001	-.116	-.294	-.212	-.085	-.136	-.030	-.007
	.042 [a,b]	.160	.009 [a,b]	.207	.996	.528	.102	.245	.643	.458	.869	.968
RCFT, immediate recall	.005	-.060	.244	.126	.208	.073	.074	.054	-.033	.034	-.022	-.008
	.979	.743	.179	.493	.253	.690	.688	.770	.859	.855	.904	.967
RCFT, delayed recall	-.036	-.090	.159	.196	.128	.000	-.062	-.042	-.239	-.063	-.158	-.289
	.844	.624	.385	.282	.487	.998	.736	.821	.189	.731	.389	.108
RCFT, recognition	-.127	.086	.169	.209	.174	.060	.211	.105	-.030	-.072	-.016	.033
	.489	.640	.356	.252	.341	.742	.246	.566	.871	.696	.929	.858
Frontal executive function												
COWAT, animal	.289	.006	.319	-.032	.085	-.216	-.113	-.220	.100	-.088	-.282	-.308
	.108	.975	.075	.864	.645	.235	.539	.225	.585	.633	.118	.086
COWAT, supermarket	.186	-.039	.319	-.173	.099	-.134	-.085	-.134	.026	-.192	-.252	-.255
	.307	.834	.075	.345	.590	.466	.645	.465	.887	.292	.165	.159
COWAT, phonemic total	.110	.080	.282	-.184	.130	.056	.048	.044	.306	.147	-.070	-.004
	.550	.663	.118	.313	.479	.760	.795	.811	.089	.424	.704	.982
Stroop test, color reading	.155	.111	.359	.200	.213	.067	.005	.025	.169	.037	-.057	-.047
	.398	.545	.043	.272	.242	.716	.977	.892	.356	.840	.758	.800
TMT-A	.405	.415	.356	.223	-.006	.060	.218	.074	.125	.098	-.294	-.224
	.022 [a,b]	.018 [a,b]	.046 [a,b]	.220	.972	.745	.231	.686	.495	.593	.102	.218
TMT-B	.066	.025	.108	.093	.134	.083	-.100	-.183	.025	-.076	-.180	-.169
	.719	.892	.557	.613	.464	.652	.586	.316	.891	.678	.325	.355
Global cognition												
MMSE	.485	.496	.284	.151	.072	.071	.127	.125	.342	.259	-.326	-.312
	.005 [a]	.004 [a]	.116	.408	.693	.701	.487	.494	.056	.153	.069	.082
CDR-SOB	-.564	-.359	-.523	-.295	-.318	-.220	-.277	-.159	-.291	-.204	.154	.154
	.001 [a]	.044 [a,b]	.002 [a]	.101	.076	.226	.125	.386	.106	.263	.401	.399

Abbreviation: LOAD, late-onset Alzheimer's disease; L, left; R, right; K-BNT, Korean version of the Boston naming test; RCFT, Rey-Osterrieth complex figure test; SVLT, Seoul verbal learning test; COWAT, controlled oral word association test; TMT-A/B, trail making test type A/B; MMSE, mini-mental status examination; CDR-SOB, clinical dementia rating-sum of boxes

Pearson's correlation analysis after adjusting intracranial volume was used and data are coefficient *r* and p value

[a] $P < 0.05$; [b] insignificant after region-wise correction for multiple comparisons

Figure S1 General linear model showing effect of global THK, FLUTE, and mean cortical thickness on the subcortical local volume

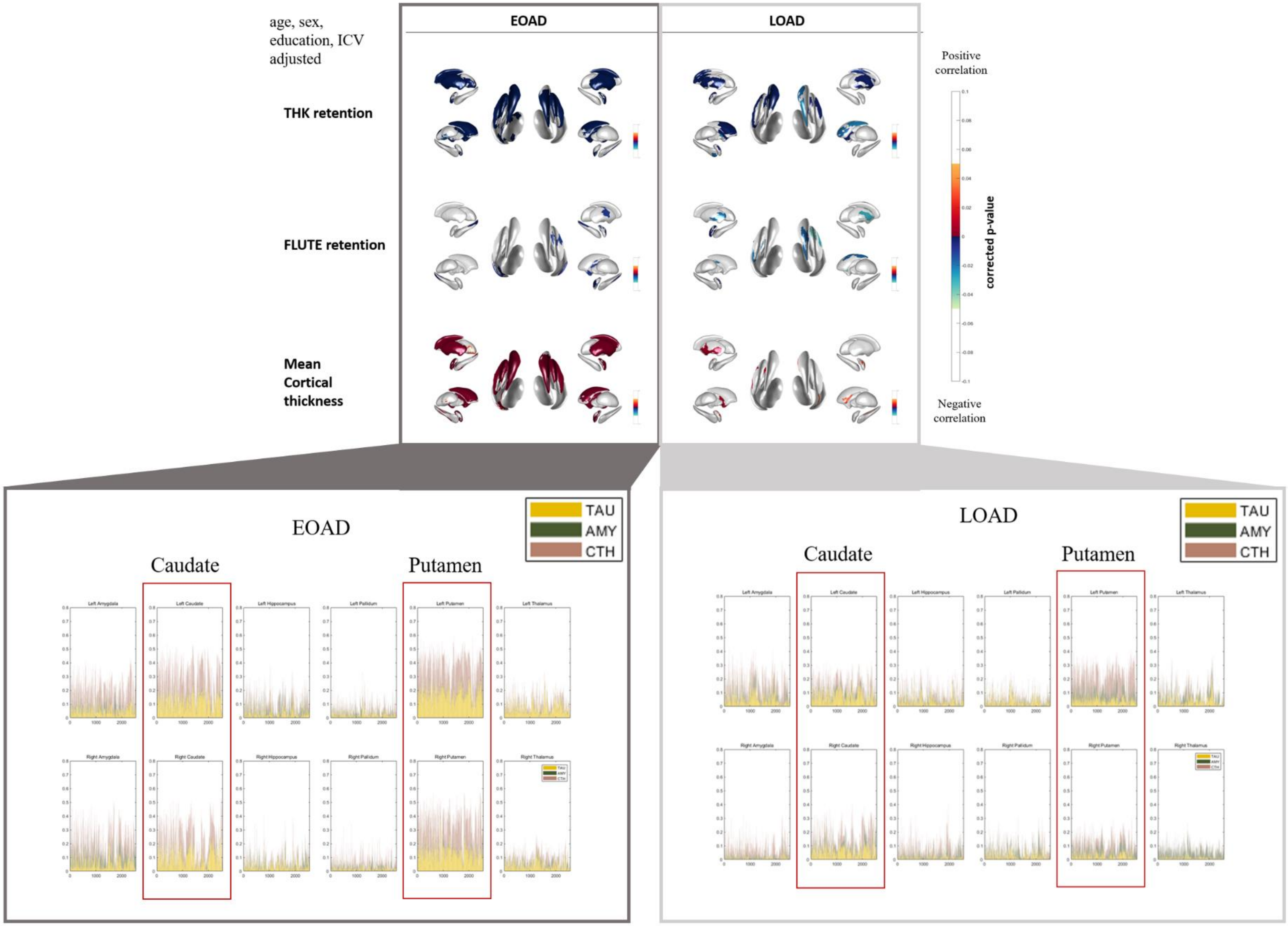

Using generalized linear model, age, sex, education and ICV were used as covariates, and the degree of tau retention, amyloid retention, and mean cortical thickness were used as predictors, and local volume values for each vertex were encoded as dependent variable. The r-squared value of the model was calculated from the predictor combinations in all cases in which a specific predictor was included in the general linear model, and the accumulated value was called relative importance. Abbreviations: EOAD, early-onset Alzheimer’s disease; LOAD, late-onset Alzheimer’s disease; ICV, intracranial volume; TAU, global tau; AMY, cortical amyloid; CTH, mean cortical thickness

Figure S2. Correlation between subcortical structure volume and THK or FLUTE.

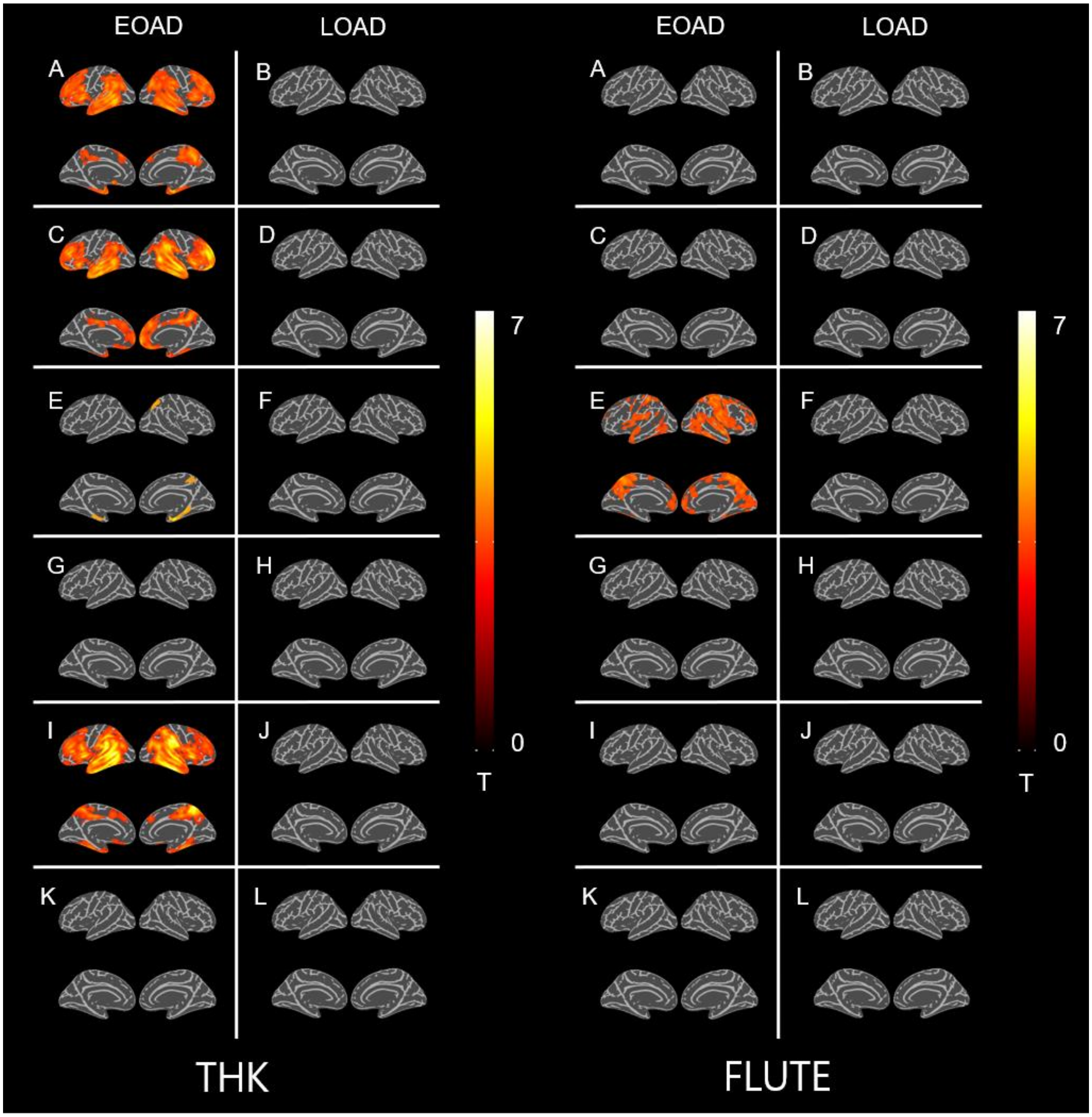

Left: Negative correlation between [18F]THK5351 SUVR and subcortical structures volume. Right: Negative correlation between [18F]flutemetamol SUVR and subcortical structures volume. A, B=amygdala; C, D=caudate; E, F=hippocampus; G,H=pallidum; I, J=putamen; K,L=thalamus. FDR correction $p < 0.05$. Adjusted for age and ICV. Voxel threshold=50

Abbreviations: EOAD, early-onset Alzheimer's disease; LOAD, late-onset Alzheimer's disease; SUVR, standardized uptake value ratio; FDR, false discovery rate; ICV, intracranial volume
