# Supplementary figures and images for: Association of Subcortical Structural Shapes With Tau, Amyloid, and Cortical Atrophy in Early-Onset and Late-Onset Alzheimer’s Disease
Source: Front Aging Neurosci. 2020 Oct 26;12:563559. doi: 10.3389/fnagi.2020.563559 (PMC7650820; doi:10.3389/fnagi.2020.563559)

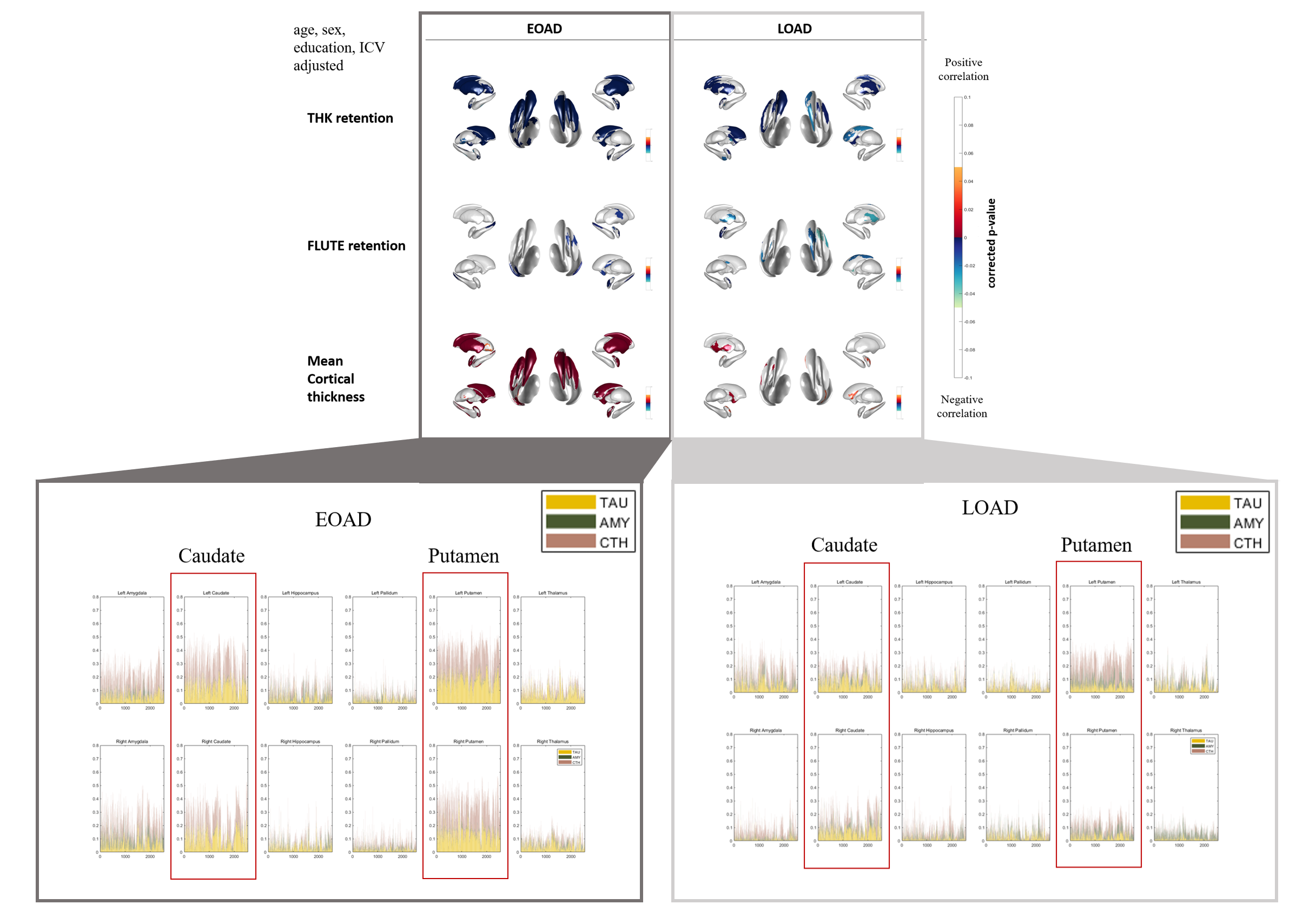

Supplement: Supplementary file 2 [file Image_1.TIF]

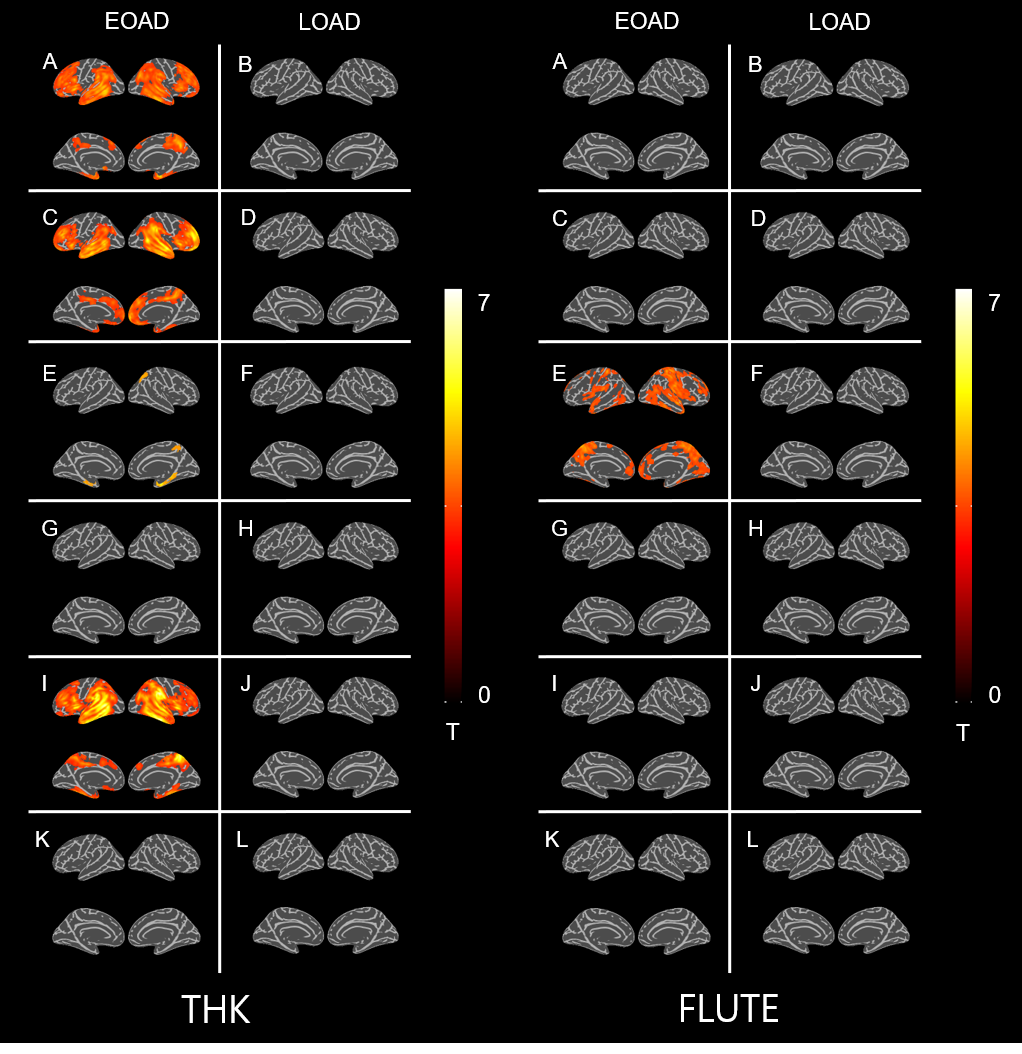

Supplement: Supplementary file 3 [file Image_2.TIF]
